# Impact of Self-Watching Double J Stent Insertion on Pain Experience of Male Patients: A Randomized Control Study Using Visual Analog Scale

**DOI:** 10.1155/2013/523625

**Published:** 2013-04-15

**Authors:** Naser S. Hussein, M. R. Norazan

**Affiliations:** ^1^Urology Department, Al-Karamah Teaching Hospital, 7 Abkar, 26047 Baghdad, Iraq; ^2^Faculty of Computer and Mathematical Sciences, Universiti Teknologi MARA (UiTM), 40450 Shah Alam, Selangor Darul Ehasn, Malaysia

## Abstract

*Objective*. To confirm safety and feasibility of double J stent insertion under local anesthesia and to assess the effect of detailed explanation and observing double J stent insertion on pain experience of male patients. *Material and Methods*. Eighty consenting males, randomized and divided prospectively into group A, who were allowed to observe DJ stent insertion, and group B, were not observed. All DJ stent insertions were done by senior urologist in operating urology room with or without fluoroscopy guidance. At the end of the procedure the vital signs and duration of the procedure were documented and patients were asked to fill unmarked 100 mm visual analogue pain scale (VAS) as soon as the surgeon leaves operating room. *Results*. Mean age of entire study group was 38.8 years; the majority of the patients had DJ stent insertion for obstructed ureteric stone, with uneventful outcomes. Postprocedural systolic blood pressure and mean pain using VAS showed statistically significant difference between groups A and B. *Conclusion*. DJ stent insertion under local anesthesia is a safe and feasible procedure. We recommended self-watching and detailed explanation to patients who underwent DJ stent insertion to reduce the pain and anxiety associated with the procedure.

## 1. Introduction

Double J (DJ) ureteric stent insertion under general anesthesia (GA) is more frequent procedure in daily work of our urology department. Furthermore this trend of practice will be at expense of inpatient beds availability and staffs dependent.

The modern cystoscopic double J ureteric stent insertion was first described in 1978, which was traditionally performed under general anesthesia on inpatients [1].

The reliance on GA for this procedure in acute setting can delay the time to stent placement, depending on staff and resources availability [2].

Moreover, the GA is not without limitations and risk especially in obese, elderly, or those with cardiovascular comorbidities. Airway trauma, swelling, vocal cord paralysis, bronchospasm, aspiration, and death from improper intubation all are reported complications of endotracheal intubation [3]. 

Despite the advancement in endourology field, studies describing the safety and feasibility of DJ stent insertion under local anaesthesia are few and did not assess the pain experience [4–7].

The concept of distraction using music, detailed explanation and viewing the procedure during endoscopies is not new.

It is used to reduce pain and anxiety during minimally invasive operation, for example, bronchoscopy, colonoscopy, colposcopy, and cystoscopy [8–11].

Recently, five randomized controlled studies regarding effects on pain of patients watching cystoscopy procedure have been published. Soomro et al., Patel et al., and Clements et al. observed a reduction in pain level in patient group allowed to view their procedure. Contradicting these findings, Cornel et al. and Kesari et al. found no difference in pain and anxiety level between two groups [8, 12–15].

Pain is a subjective feeling; however, it can be measured indirectly using visual analog scale (VAS) and autonomic changes such as tachycardia, hypertension, lacrimation, and diaphoresis which all can be established signs of pain or inadequate analgesia [16]. 

The objectives of this study are to confirm safety and feasibility of DJ stent insertion under local anesthesia (LA) and to assess the effect of detailed explanation and observing of DJ stent insertion in level of pain experience in male patients, using visual analog scale (VAS) and vital signs changes (pulse rate, systolic and diastolic blood pressure) before and after the procedure.

VAS is a 100 mm horizontal line that can be used to quantify pain in continuous scale of 0 to 10. It is easy to use and requires little written language [17]. 

## 2. Materials and Methods

This study was conducted at a urology department of a teaching hospital after getting an ethical committee approval.

Over a period of six months, from January to July 2012, all males above 18 years old who attended urinary stone disease clinic and have indications for DJ insertion (intractable renal pain or ureteric colic, fever and pyuria, moderate to severe degree of hydronephrosis, preexternal shock wave lithotripsy-ESWL-renal stone more than 2 cm, and anuria due to urinary stone) were included in this study.

Exclusion criteria were history of previous cystoscopy, clinical evidence of urethral stricture, psychiatric illness, inability to understand the procedure or the questionnaires, bladder outlet obstruction, stent exchanges, and patient requesting general anaesthesia.

All DJ ureteric stents were inserted as temporary measurement while waiting for definitive treatment 4–6 weeks later according to operation room availability.

Eighty consenting subjects were randomized by drawing lots to observe or not to observe their DJ stent insertion.

They were grouped into group A (those patients who were to view their procedure) and group B (those patients who were not allowed to view their procedure).

All patients received brief explanation from medical officer about the procedure in outpatient clinic then admitted to hospital as day case.

For group A, a video monitor was placed so that both patient and operating urologist could see the procedure. For group B, the monitor was positioned so that only the operating surgeon could visualize the procedure and not the patient. 

The procedure was performed in urology operating room in lithotomy position by a senior urologist with or without fluoroscopy guidance, where position of DJ stent was checked postoperatively by kidney, ureter, and bladder X-ray (KUB-X-ray).

A peripheral venous line was fixed and prophylactic intravenous antibiotic was given.

After positioning of the patient and scrubbing with povidone-iodine solution and standard draping, 2% of lidocaine gel was instilled in urethra. 

DJ stent was inserted in all subjects in standard fashion using 20 F storz rigid cystoscope with 30 degree lens with favorable outcomes and there is no intra- or postoperative complications.

Patients' preprocedure pulse rate, systolic and diastolic blood pressure, and procedure time were recorded.

Immediately after urologist left the operating room, a medical officer who was blinded to the details of the study collected data on vital signs and asked patients to record their pain experience using a 100 mm unmarked VAS.

We categorized the pain as mild, moderate, and severe. No pain was defined as a score 0, mild pain as a score 1–3, moderate pain as a score 4–7, and severe pain as a score 8–10.

All patients were informed about the possible infective consequences of the procedure and given ready access to telephone advice or hospital admission if required. 

Descriptive statistics were used to summarize the results. Chi-square test was used to check for a relationship between pain categories and patient group types. Independent *t*-tests were used to determine the mean differences in the level of pain and both pre- and postprocedure vital sign changes between the two groups. Finally, paired *t*-tests were used for the assessment of mean differences between pre- and postprocedure vital signs for each group. In this study, a test with *P* value < 0.05 was considered statistically significant. 

## 3. Results and Analysis

Table 1 demonstrates that, the mean patient age was 38.8 years. The average duration of procedure was 5.35 ± 0.87 minutes. The number of patients who had DJ insertion for ureteric stone was 46 (57.5%) of the total cases, renal stones in 21 cases (26.25%), followed by the anuria 13 cases (16.3%).

Using a scale of 0–10, the mean pain (standard deviation) score on VAS was found to be 3.91 ± 3.12. Category-wise, 14 (17.5%) patients did not experience pain during the procedure. On the other hand, 27 (33.8%) of the patients experienced mild pain, 28 (35%) patients experienced moderate pain, and only 11 (13.8%) experienced severe pain. We performed a chi-square test to determine whether there is a relationship between the pain categories and patient group types. The chi-square test produces a *P* value = 0.000 < 0.05, implying that the pain categories experienced by the patients have an association with the group that they belong to.

To confirm these findings, we performed an independent *t*-test to determine whether the mean of pre- and postprocedure vital signs is significantly different for groups A and B. The independent *t*-test was also done to check for the significant difference in mean age and duration of the procedure. 

Table 2 shows the results of the tests. Note that any test with the *P* value > 0.05 indicates that the *t*-test is statistically not significant.

We observed from Table 2 that the mean age and duration of the procedure were not much different for the two groups. The pulse rate and systolic and diastolic blood pressures before the procedures were also found to be comparable for both groups of patients. 

However, the systolic blood pressure after the procedure and the mean pain using VAS are statistically and significant different (with *P* value < 0.05). These findings confirm that those patients who could view the procedure experience less pain as compared to those who did not view the procedure. The mean pain score experienced by the patients from group B (who could not view the procedures) is almost four times higher than the mean pain score of group A (who did view the procedures).

Fourteen patients from group A experienced no pain at all, compared to none from group B. 

Majority of patients from group B experienced moderate pain, but majority from group A only experienced mild pain. 

Ten patients from group B experienced severe pain as compared with only one patient in group A. 

Table 2 shows that majority of patients from group B, who did not view the procedures, were in severe and moderate pain categories. In contrast, majority of patients in group A who did view the procedures were in the categories of mild or no pain at all. 

Tables 3 and 4 display results of analysis for comparing the means of vital sign changes before and after the procedures for individual groups A and group B, respectively.

Generally, we notice that for both groups, the vital sign means after the procedure are higher than the vital sign means before the procedures. For group B, the mean of systolic and diastolic blood pressure increases after the postprocedure. The results of paired tests confirm the significant increase in the means of systolic and diastolic blood pressures, but no significant increase in pulse rates. However, for group A, only diastolic blood pressure mean is significantly higher after the postprocedure.

## 4. Discussion

Demands on limited inpatient bed and ancillary resources, operating theater, saving money and time are increasing and any opportunity to reduce those demands with use of outpatient procedures is always interesting.

DJ stent is generally inserted in operating room with or without fluoroscopic guidance using either flexible or rigid cystoscopy as both tolerated well and no significant difference in outcomes as reported by Sivalingam et al. [2].

Local or general anesthesia is still an issue of discussion; despite a higher rate of side effects than LA or regional anesthesia, general anesthesia remains the most commonly used anesthetic technique for ambulatory surgery [18]. 

Pain during cystoscopy can be influenced by type, volume, time, and temperature of lubricant used, viewing and detailed explanation of the procedure [19–22]. 

However none of these studies were successful in changing the pain and anxiety during cystoscopy. 

Moreover Cornel et al. demonstrated in their study that history of cystoscopy is unlikely to affect the pain experience during the procedure [15].

Previously published studies demonstrated no effect of watching cystoscopy on pain. On the opposite side, other studies showed that viewing the procedure has effect on pain experience of patients, as pain was measured by VAS [8, 12–15]. 

All previously published studies tested the pain during diagnostic, followed up cystoscopy and minor therapeutic procedure (DJ removal), and assessed the feasibility of DJ stent insertion under LA as separated topic. To our best knowledge this work has assessed feasibility of DJ stent insertion under LA and tested the effect of detailed explanation and real-time video monitoring of the procedure on pain experience as one subject.

Visual analogue scale (VAS) is a valid tool with good responsivity and acceptability; it has been used extensively in the medical literature. 

Clinically VAS can be used for pain, nausea, fatigue, and sleep quality measurement, with no clinical significant difference between severity of pain being experienced and VAS pain score [23–27].

Our study shows that patients who had the chance to view the procedures experience less pain compared to those who did not.

Meanwhile, we believe that those patients of group A, had moderate and severe pain, as further improvement in decreasing the pain of this group might be not possible due to overall minimal anxiety in accordance with Kobayashi et al. findings [28].

Overall, there is an increase in the means of the vital signs after the procedure, but it seems that group B has experienced a more significant increase if compared to group A. 

In addition there was statistically significant difference in postprocedural systolic blood pressure between groups; however these changes in blood pressure may be from clinical point of view insignificant, as Soomro et al. reported that minor changes in vital signs may be related to other factors besides procedure-related discomfort [12].

## 5. In Conclusion

DJ stent insertion under local anesthesia is safe and feasible. We recommended self-watching and detailed explanation to patients who underwent DJ stent insertion to reduce the pain and anxiety associated with the procedure.

## Figures and Tables

**Table 1:** Basic procedural and demographic characteristics of the entire patient group.

Mean age, y (range)	38.83 (18–78)
Mean duration of procedure, min	5.35 ± 0.87
Indication for DJ insertion, *n* (%):	
Ureteric	46 (57.5)
Renal	21 (26.25)
Anuria	13 (16.3)
Pain score, mean ± SD	3.91 ± 3.12
Pain, by category:	
No pain (VAS scale = 0), *n* (%)	14 (17.5)
Mild pain (VAS scale = 1–3), *n* (%)	27 (33.8)
Moderate pain (VAS scale = 4–7), *n* (%)	28 (35.0)
Severe pain (VAS scale = 8–10), *n* (%)	11 (13.8)

**Table 2:** Comparison of pain (based on visual analog scale) and vital signs in the 2 patient groups.

	Group A	Group B	*P* value
Age, y, mean ± SD	38.13 ± 14.72	39.53 ± 14.98	0.675
Duration of procedure, min	5.34 ± 0.89	5.36 ± 0.87	0.899
Preprocedure, mean ± SD:			
Systolic blood pressure, mm Hg	125.80 ± 18.44	132.40 ± 21.524	0.145
Pulse, beats/min	82.25 ± 5.969	80.93 ± 7.072	0.369
Diastolic blood pressure, mm Hg	78.08 ± 7.502	78.38 ± 8.369	0.866
Postprocedure, mean ± SD:			
Systolic blood pressure, mm Hg	126.63 ± 15.590	135.90 ± 20.348	0.025*
Pulse, beats/min	83.38 ± 5.077	81.90 ± 6.543	0.263
Diastolic blood pressure, mm Hg	79.50 ± 6.872	80.28 ± 7.786	0.638
Mean pain score (VAS)	1.40 ± 1.932	6.43 ± 1.752	0.000*
Pain, by category:			
No pain	14	0	
Mild pain	22	5	
Moderate pain	3	25	
Severe pain	1	10	

*A *P* value < 0.05 is considered statistically significant.

**Table 3:** Test for the difference in means of pre- and postprocedures for group A.

	Preprocedure mean ± SD	Postprocedure mean ± SD	*P* value
Systolic blood pressure, mm Hg	125.80 ± 18.44	126.63 ± 15.590	0.498
Pulse, beats/min	82.25 ± 5.969	83.38 ± 5.077	0.150
Diastolic blood pressure, mm Hg	78.08 ± 7.502	79.50 ± 6.872	0.023*

*A *P* value < 0.05 is considered statistically significant.

**Table 4:** Test for the difference in means of pre- and postprocedures for group B.

	Preprocedure mean ± SD	Postprocedure mean ± SD	*P* value
Systolic blood pressure, mm Hg	132.40 ± 21.524	135.90 ± 20.348	0.000*
Pulse, beats/min	80.93 ± 7.072	81.90 ± 6.543	0.061
Diastolic blood pressure, mm Hg	78.38 ± 8.369	80.28 ± 7.786	0.010*

*A *P* value < 0.05 is considered statistically significant.
